# Assessing the Efficacy of Citrus Aurantifolia Extract on Smear Layer Removal with Scanning Electron Microscope

**Published:** 2012-06-01

**Authors:** Behnam Bolhari, Mohammad Reza Sharifian, Mohsen Aminsobhani, Hamid Reza Monsef Esfehani, Pardis Tavakolian

**Affiliations:** 1. Department of Endodontics, School of Dentistry/Dental Research Center, Tehran University of Medical Sciences, Tehran, Iran; 2. Department of Pharmacology, Pharmacy School, Tehran University of Medical Sciences, Tehran, Iran; 3. Dentist, Tehran, Iran

**Keywords:** Citrus Aurantifolia, Irrigation, Root Canal Therapy, Sem, Smear Layer

## Abstract

**Introduction:**

The purpose of this study was to determine the effects of citrus aurantifolia (CA) extract on smear layer removal in different parts of root canals.

**Materials and Methods:**

Thirty-nine single-rooted human teeth were randomly divided into three experimental (n=12) and one control (n=3) groups. Teeth were instrumented using MTwo rotary instruments. Root canals were irrigated with NaOCl during instrumentation. The canals in each group were irrigated with one of the following: completed mixture of citrus aurantifolia extracts, 17% EDTA, and alcoholic extract of CA. Distilled water was used for the control group. The irrigants were left within the canal for 20 minutes, and then rinsed with normal saline solution. Teeth were subsequently split longitudinally into 2 halves, and the canals were examined by a scanning electron-microscope. Cleanliness was evaluated using a five point scoring system.

**Results:**

Statistical significant difference was found between groups (P<0.05). The smear layer was more effectively removed with 17% EDTA compared to alcoholic CA extract. However, they were both able to remove the smear layer in the coronal segment. Completed CA extract removed more smear layer in coronal and middle parts compared with the alcoholic extract (P=0.001); however, there was no significant difference in the apical part.

**Conclusion:**

Both of the alcoholic and completed mixtures of citrus aurantifolia extracts were not able to effectively remove smear layer compared with 17% EDTA during root canal therapy.

## Introduction

Root canal cleaning means removing all potential irritants such as bacteria and their byproducts, organic/inorganic debris, vital and necrotic pulp tissues, as well as blood. Acceptable cleaning of the root canal can be achieved through irrigation and instrumentation [1]. One of the main purposes of cleaning and shaping the canal system is to maintain long-term success after the root canal therapy (RCT) [2]. In fact, the aim of RCT is to obtain bacteria/debris-free clean root canals as well as creating a proper seal through the root canal obturation [3][4].

Smear layer is left on the canal walls after instrumentation. This layer consists of organic and inorganic particles of dentin, vital and necrotic pulp tissue, microorganisms and blood cells [5]. However there is some controversy regarding the removal of smear layer in dentinal tubules [6]. Latest evidence showed that the smear layer inhibits the penetration of antimicrobial irrigants and medication into the dentinal tubules [7][8]. Therefore, for closer adherence of obturants to the root canal wall and to reduce the apical as well as coronal microleakage, the smear layer should ideally be removed [9][10].

Various irrigants are used for root canal treatment [6]. Sodium hypochlorite (NaOCl), one of the most popular irrigants in endodontics, has strong antimicrobial activity and organolytic effects, however it cannot remove smear layer [11][12][13]. The smear layer mainly consists of inorganic substances which are soluble in acids. Various types of acids like Ethylene-Di-Amino-Tetra-acetate (EDTA), Citric acid, tannin, and poly acrylic acid are suitable chemicals for smear layer removal [14]. Goldman et al. confirmed that the a final flush with 17% EDTA followed by NaOCl will completely remove the smear layer [15][16].

EDTA is mostly used for smear layer removal; some studies have shown that it cannot effectively remove the smear layer in the apical third of the root canal [12][17]. However, irrigation with EDTA followed by NaOCl could demineralize the dentine and produce erosions in coronal as well as the middle part of the root canal [15][18].

Recently, the citric acid has been suggested for smear layer removal. Citric acid is a weak organic acid, which belongs to the chelating agents category [19]. One study found that 10% citric acid and 2.5% NaOCl are effective solutions for smear layer removal [20]. Di Lenarda et al. showed no significant differences in smear layer removal between citric acid and EDTA [19].

In this study, we chose citrus aurantifolia(lime juice) extract as the final irrigant, because it consists of citric acid along with an added antimicrobial feature. Lime juice contains 88% water, 6-8% citric acid, 2% potassium citrate and calcium, 0.4-0.6% and other substances. Because citrus aurantifolia extract has citric acid, it is able to remove the smear layer and open the dentinal tubules [21].

We aim to evaluate the efficacy of citrus aurantifolia as smear layer removing agent using a scanning electron-microscope (SEM).

## Materials and methods

Thirty nine single-rooted teeth were selected and they were included single canals without any caries and resorption.

All the teeth were radiographically assessed to ensure absence of calcification or resorption in the root canals. The teeth crowns were cut at the CEJ by a disk creating average root length of 14 to17 mm. The roots were randomly divided into three experimental groups of A, B, C (n=12) and one control group (n=3).

A #15 k-file was used to determine the working length. All roots were instrumented up to a #40 Mtwo rotary file (VDW, Munich, Germany) at the apical part, and 2 mL NaOCl (5.25%) was used as irrigant between each files. To remove the smear layer the following four different final irrigation methods were used [17]:

Group A: 1 mL 17% EDTA (Apadana Tak Co, Tehran, Iran) for 20 min.

Group B: 1 mL complete extraction of citrus aurantifolia for 20 min.

Group C: 1 mL alcoholic extraction of citrus aurantifolia for 20 min.

Group D: 1 mL Distilled water for 1 min.

The reason we chose 20 minute irrigation was that Sharifian et al. had evaluated the antimicrobial effect of citrus aurantifolia extract in this time period [22].

After 20 minutes, the roots were rinsed with normal saline and then were longitudinally grooved on the external surface with a cutting disk. Afterwards, the Afterwards, the roots were split in two halves with a chisel. Half of the root was placed in 2% glutaraldehyde solution for 24 h The fixed specimens were rinsed three times with a sodium cacodylate buffered solution (0.1 M, PH 7.2), then incubated in osmium tetroxide for 1 hour followed by dehydrated with ascending, concentrations of ethyl alcohol (30-100%), and placed in a desiccator for at least 24 h Each specimen was mounted on aluminum stub and coated with 25 µm of gold-palladium and examined under a SEM.

The magnification of all the photomicrographs was 2000X and all specimens were observed and examined in coronal, middle and apical parts of their root canal walls.

Cleanliness was evaluated using a five-point scoring system codified by Schafer and Lohmann as below [23]:

Score 1: Clean root canal wall, only few small debris particles.

Score 2: Few small agglomerations of debris.

Score 3: Many agglomerations of debris covering less than 50% of the root canal wall.

Score 4: More than 50% of the root canal wall covered by debris.

Score 5: Complete or nearly complete root canal wall covered by debris.

Three researchers observed the SEM images independently (double-blind). All the data were analyzed using by Kruskal-Wallis and Dunn test.

## Results

**Figure 1:**
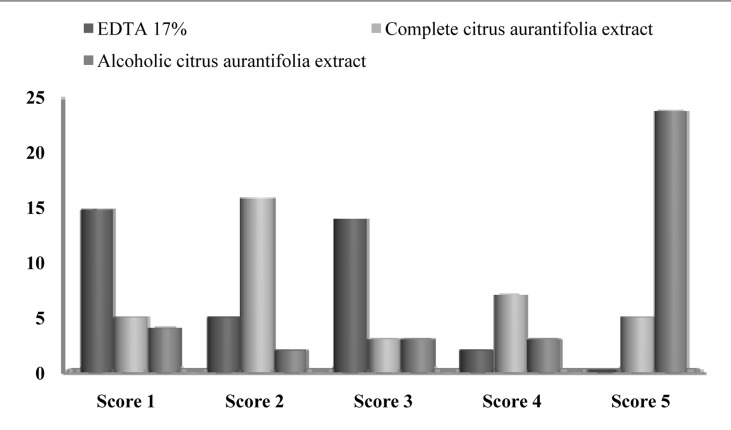
Distribution of specimens (number) in each cleanliness score (1-5; amount of smear layer removal) in the coronal part of the root for the three experimental groups

**Figure 2:**
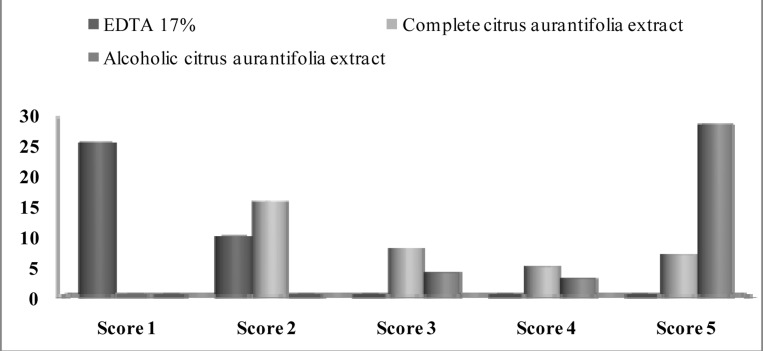
Distribution of specimens (number) in each cleanliness score in middle part of the root in all groups

**Figure 3:**
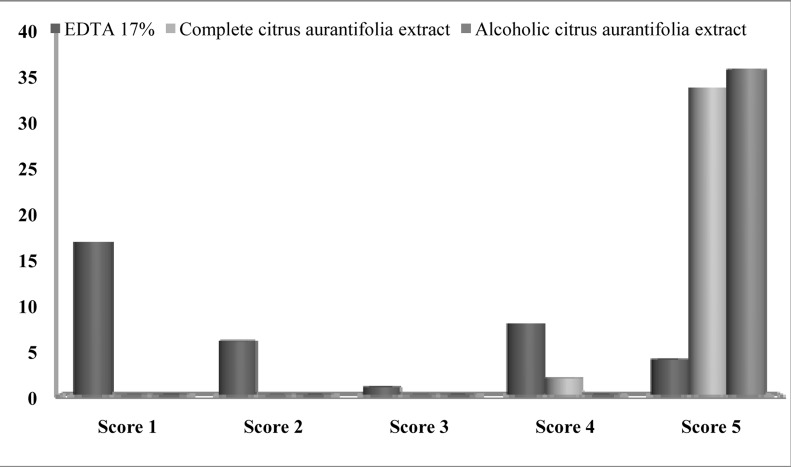
Distribution of specimens (number) in each cleanliness score in apical part of the root in all groups

There was more than 90% agreement on scoring the images between the researchers. After group discussion, all three researchers came into complete agreement with regard to scoring. The sum of the scores of the three evaluators is shown in Figure 1, Figure 2 and Figure 3.

**Figure 4:**
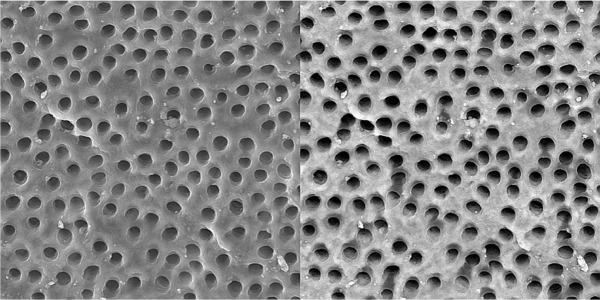
Effects of 17% EDTA on coronal third of the root canal (SEM; original magnification×2000)

**Figure 5:**
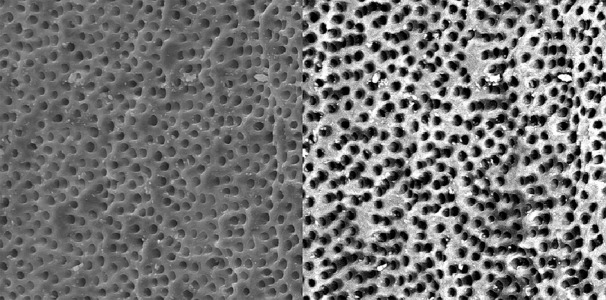
Effects of 17% EDTA on middle third of the root canal (SEM; original magnification×2000)

**Figure 6:**
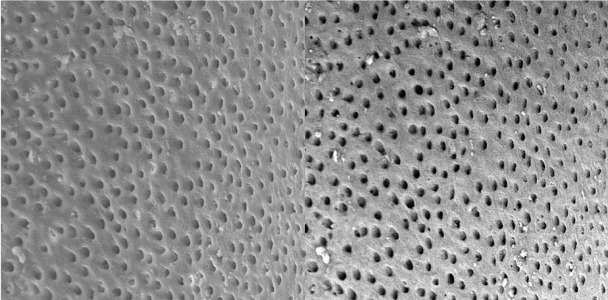
Effects of 17% EDTA on apical third of the root canal (SEM; original magnification ×2000)

Group A. 17% EDTA:

Specimens irrigated with 17% EDTA (final irrigant) showed cleaned root canal walls in the coronal and middle parts (Figure 4, Figure 5); the apical part was only partially clean Figure 6.

**Figure 7:**
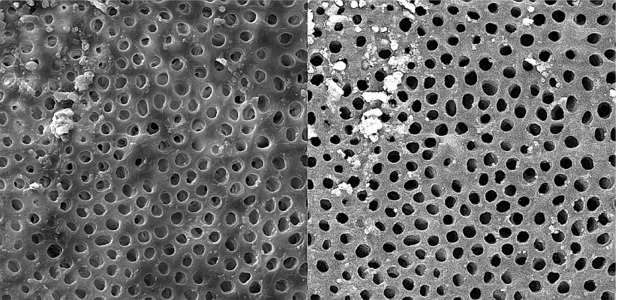
Effects of complete citrus auranatifolia extract on coronal third of the root canal (original magnification ×2000)

**Figure 8:**
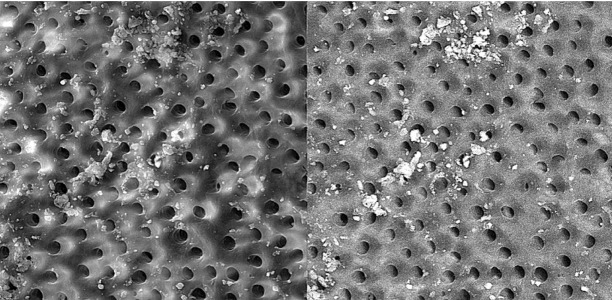
Effects of complete citrus auranatifolia extract on middle third of the root canal (original magnification ×2000)

**Figure 9:**
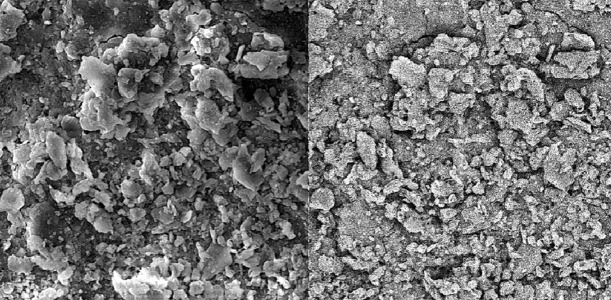
Effects of complete citrus auranatifolia extract on apical third of the root canal (original magnification ×2000)

Group B. Completed mixture of CA extract:

In these specimens, the smear layer was partially removed in coronal and middle parts of the root canal (Figure 7, Figure 8); however, the smear layer in apical part was not removed (Figure 9).

**Figure 10:**
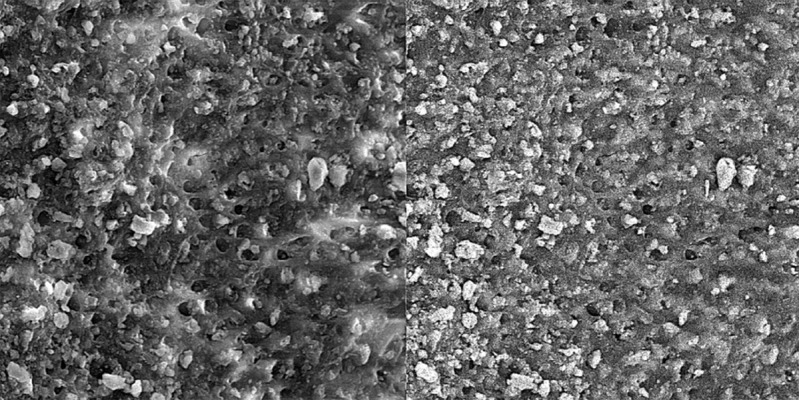
Effects of alcoholic citrus auranatifolia extract on coronal third of the root canal (original magnification ×2000)

**Figure 11:**
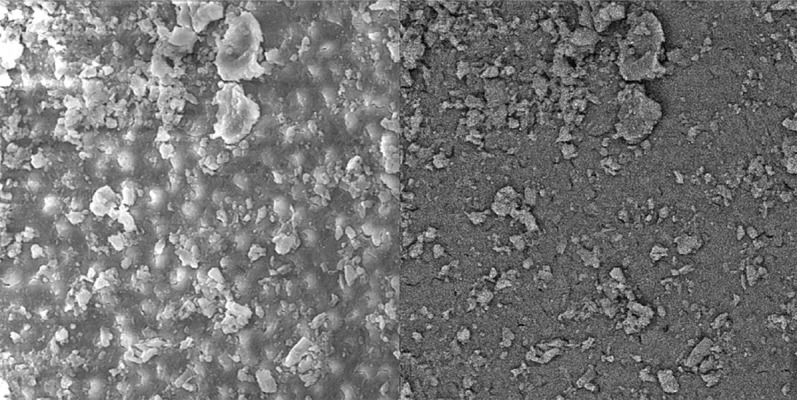
Effects of alcoholic citrus auranatifolia extract on middle third of the root canal (original magnification ×2000)

**Figure 12:**
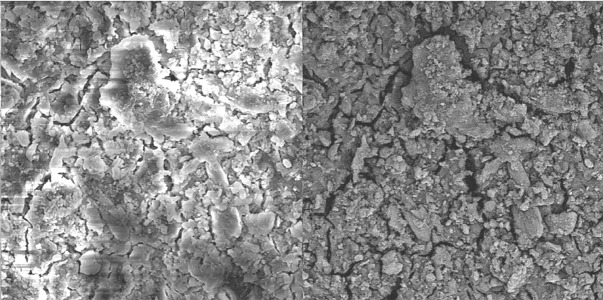
Effects of alcoholic citrus auranatifolia extract on apical third of the root canal (original magnification ×2000)

Group C. Alcoholic CA extract:

Alcoholic citrus aurantifolia extract was not effective enough to remove the smear layer in all three parts of the root canal (Figure 10, Figure 11 and Figure 12).

**Figure 13:**
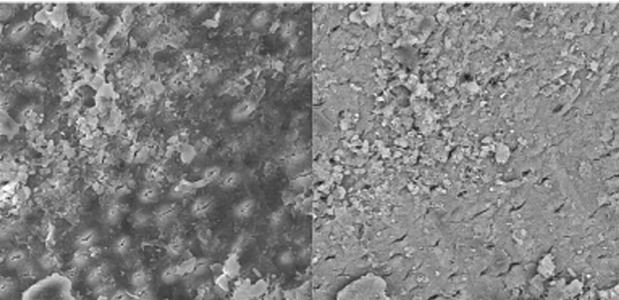
Effects of NaOCl on coronal third of the root canal (original magnification ×2000)

**Figure 14:**
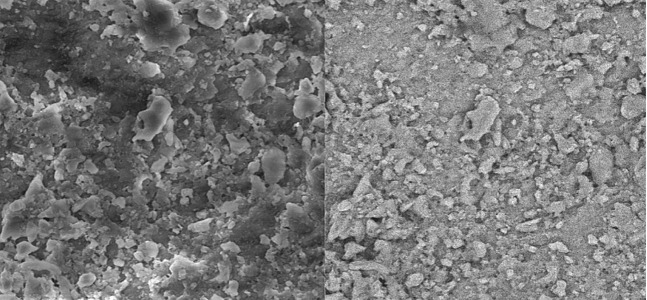
Effects of NaOCl on middle third of the root canal (original magnification ×2000)

**Figure 15:**
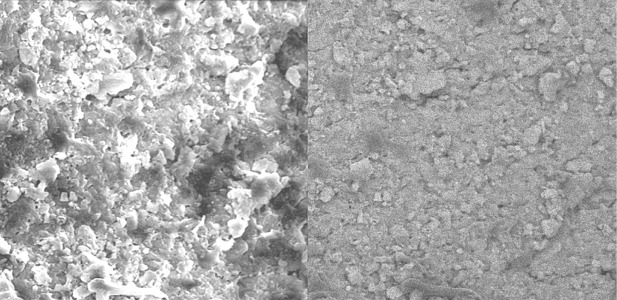
Effects of NaOCl on apical third of the root canal (original magnification ×2000)

Group D. Distilled water:

The smear layer was not removed by distilled water (Figure 13, Figure 14 and Figure 15).

The statistical analysis showed there was a significant difference between 17% EDTA and complete citrus aurantifolia extract (P=0.017) in the SEM analysis. In fact the efficacy of 17% EDTA was superior to the completed mixture of CA extracts, especially in apical and middle parts of the root canal wall. However there was no significant difference between them in the coronal segments (P>0.05).

There was a significant difference between 17% EDTA and alcoholic CA extract (P=0.002) in all three parts of the root canal. The efficacy of completed mixture of CA extracts was better than alcoholic CA irrigant in middle and coronal parts.

## Discussion

The smear layer is formed during root canal instrumentation consisting of dentin, bacteria, odontoblastic processes, necrotic and vital pulp tissues [24]. The smear layer can compromise coronal and apical microleakage, as well as the long term success of endodontic treatment [25].

We chose citrus aurantifolia extract for this study as it contains 6-8% citric acid (a chelating agent) in addition to its antimicrobial effects [21][22]. Specimens were irrigated for 20 minutes for maximum antimicrobial effect [22].

Previous studies indicated that 17% EDTA removes the smear layer in all three segments of the root canal, contradicting our study [2][26].

In this study, the results showed that 17% EDTA is able to remove the smear layer completely only in the coronal and middle parts of root canal. This concurs with Takeda et al. [17] and Parbhu et al. [24]. Their results also showed that 17% EDTA was not able to produce the expected smear-free surfaces in the apical part of the canal. Firstly, the apical part was less accessible than the coronal and middle parts for deeper penetration of EDTA. The second reason may be due to the diameter of the needle which may be too large to access the apical segment.

There was a significant difference between 17% EDTA and completed mixture of CA extracts in removing the smear layer from apical and middle parts of root canal. However there was no statistical difference in the coronal part; both were effective in removing the smear layer coronally.

Takeda et al. [17], Mancini et al. [27] have proved that citric acid with 6% and 42% concentration, respectively, were not able to remove the smear layer in the apical and middle parts. Our results disagree with other studies that have indicated that citric acid (with 7% and 10% concentration) removes the smear layer in all parts of the root canal. However, the concentration of citric acid used in their study was higher [2][26].

There was a significant difference between alcoholic citrus aurantifolia and 17% EDTA in removing the smear layer. The results showed that alcoholic citrus aurantifolia was not able to remove the smear layer in all three parts of the root canal.

One possible explanation could be due to certain properties, such as the other chemicals in completed CA extract or the low concentration of 6% to 8% citric acid in the irrigant. The CA extract may not have been able to penetrate deep into the apical part of the root canal (because of high surface tension). A higher surface tension requires greater pressure when injecting the liquid during the irrigation of the root canal. Also alcoholic citrus aurantifolia extract contains a very small amount of citric acid, so it was not able to remove the smear layer.

## Conclusions

Based on the results of this study, the completed mixture and alcoholic citrus aurantifolia extracts were not able to remove the smear layer completely. EDTA (17%) seems to be a more effective chelating agent within root canals.
